# Supplementary material for: Progressive Motor and Non-Motor Symptoms in Park7 Knockout Zebrafish
Source: Int J Mol Sci. 2023 Mar 29;24(7):6456. doi: 10.3390/ijms24076456 (PMC10094626; doi:10.3390/ijms24076456)
Supplement: Supplementary file 1 [file ijms-24-06456-s001.zip › Supplementary Figure, S1.pdf]

Figure S1

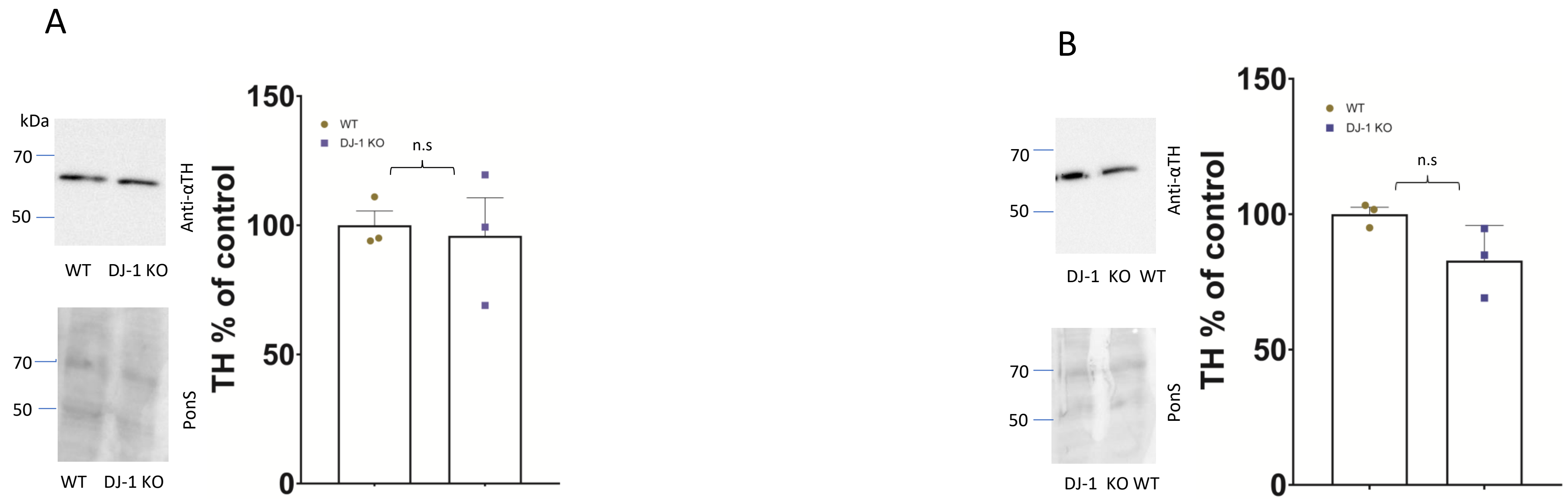

Figure S1. Tyrosine hydroxylase levels are not altered at the early adult stage. Western blots of tyrosine hydroxylase (TH) expression and densitometric quantification from 7 (A) and 10 (B) months of wild type and DJ-1 KO brains. Values are the mean ± SEM (n=3). Expression levels were normalized using PonceauS staining. n.s, non-significant ($p>0.05$) using Student's two-sample t-test.
