# Supplementary figures and images for: Progressive Motor and Non-Motor Symptoms in Park7 Knockout Zebrafish
Source: Int J Mol Sci. 2023 Mar 29;24(7):6456. doi: 10.3390/ijms24076456 (PMC10094626; doi:10.3390/ijms24076456)

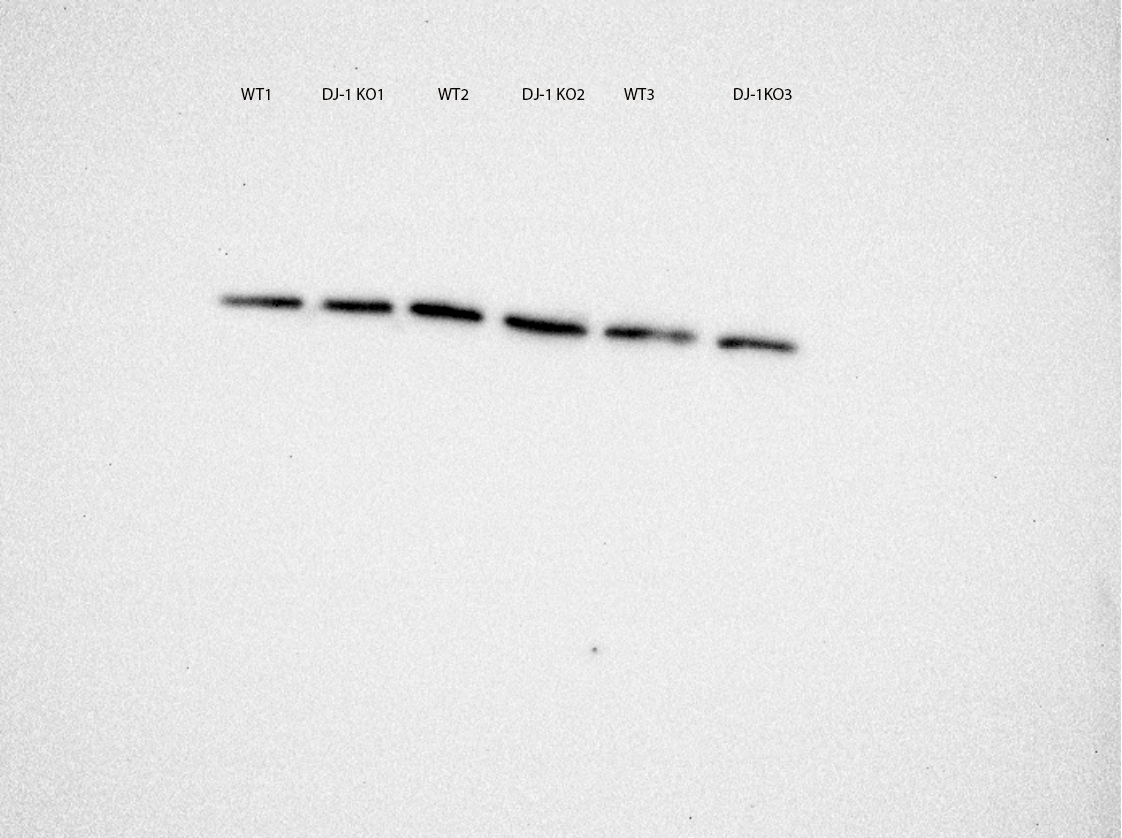

Supplement: Supplementary file 1 [file ijms-24-06456-s001.zip › Supplementary Figure, S2.tif]

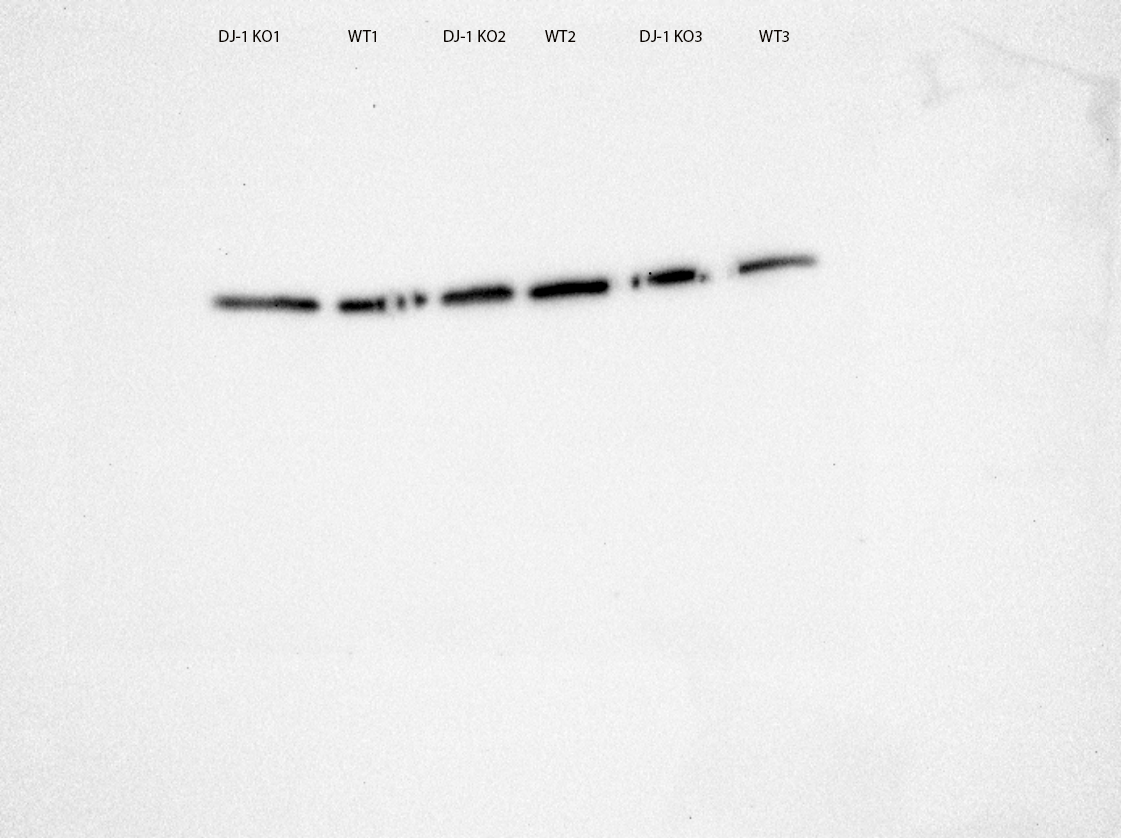

Supplement: Supplementary file 1 [file ijms-24-06456-s001.zip › Supplementary Figure, S3.tif]

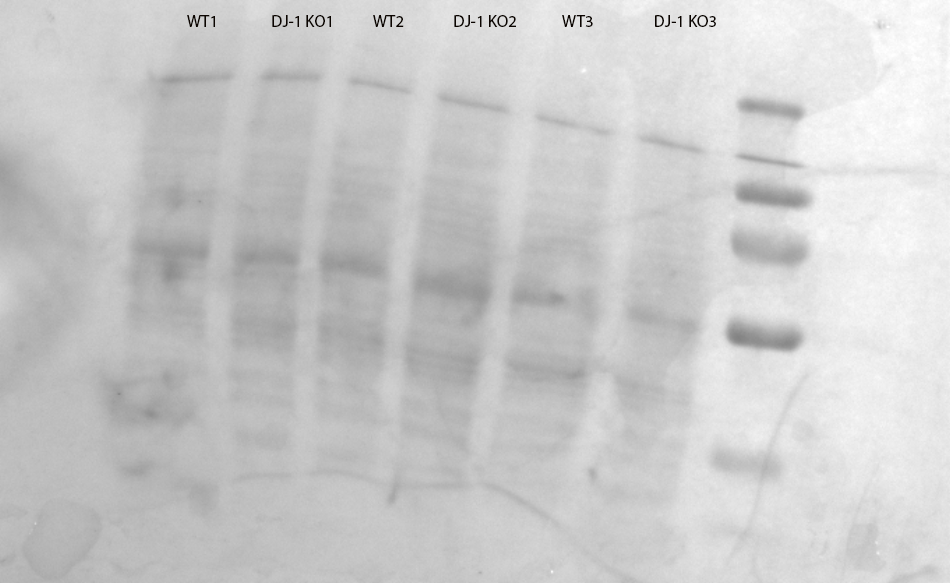

Supplement: Supplementary file 1 [file ijms-24-06456-s001.zip › Supplementary Figure, S4.tif]

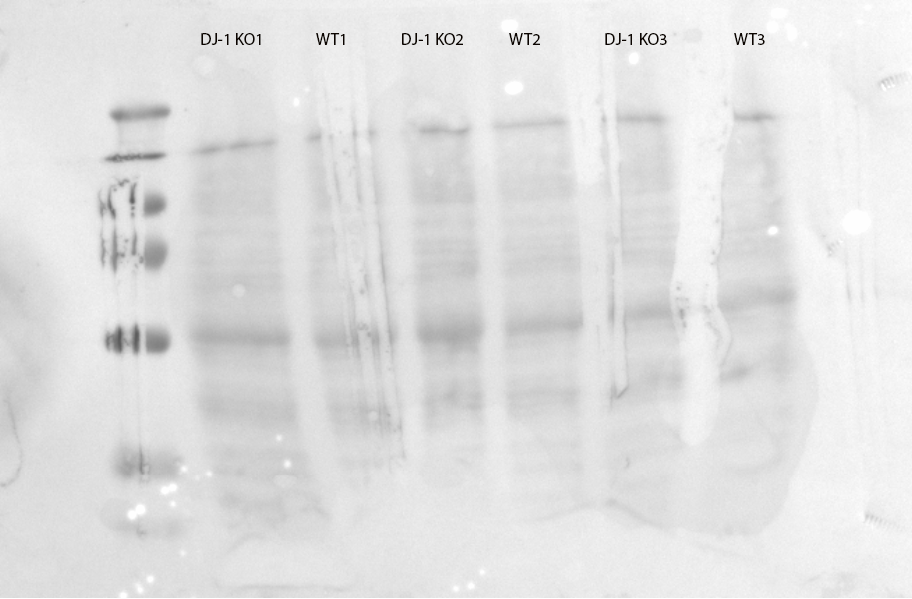

Supplement: Supplementary file 1 [file ijms-24-06456-s001.zip › Supplementary Figure, S5.tif]
